# Dietary exposure to soy or whey proteins alters colonic global gene expression profiles during rat colon tumorigenesis

**DOI:** 10.1186/1476-4598-4-1

**Published:** 2005-01-11

**Authors:** Rijin Xiao, Thomas M Badger, Frank A Simmen

**Affiliations:** 1 Arkansas Children's Nutrition Center, 1120 Marshall Street, Little Rock, AR, 72202, USA; 2 Department of Physiology and Biophysics, University of Arkansas for Medical Sciences, Little Rock, AR, 72202, USA

**Keywords:** colon cancer, soy, whey, gene expression profiling, neuro-endocrine, microarray, rat

## Abstract

**Background:**

We previously reported that lifetime consumption of soy proteins or whey proteins reduced the incidence of azoxymethane (AOM)-induced colon tumors in rats. To obtain insights into these effects, global gene expression profiles of colons from rats with lifetime ingestion of casein (CAS, control diet), soy protein isolate (SPI), and whey protein hydrolysate (WPH) diets were determined.

**Results:**

Male Sprague Dawley rats, fed one of the three purified diets, were studied at 40 weeks after AOM injection and when tumors had developed in some animals of each group. Total RNA, purified from non-tumor tissue within the proximal half of each colon, was used to prepare biotinylated probes, which were hybridized to Affymetrix RG_U34A rat microarrays containing probes sets for 8799 rat genes. Microarray data were analyzed using DMT (Affymetrix), SAM (Stanford) and pair-wise comparisons. Differentially expressed genes (SPI and/or WPH vs. CAS) were found. We identified 31 induced and 49 repressed genes in the proximal colons of the SPI-fed group and 44 induced and 119 repressed genes in the proximal colons of the WPH-fed group, relative to CAS. Hierarchical clustering identified the co-induction or co-repression of multiple genes by SPI and WPH. The differential expression of I-FABP (2.92-, 3.97-fold down-regulated in SPI and WPH fed rats; P = 0.023, P = 0.01, respectively), cyclin D1 (1.61-, 2.42-fold down-regulated in SPI and WPH fed rats; P = 0.033, P = 0.001, respectively), and the c-neu proto-oncogene (2.46-, 4.10-fold down-regulated in SPI and WPH fed rats; P < 0.001, P < 0.001, respectively) mRNAs were confirmed by real-time quantitative RT-PCR. SPI and WPH affected colonic neuro-endocrine gene expression: peptide YY (PYY) and glucagon mRNAs were down-regulated in WPH fed rats, whereas somatostatin mRNA and corresponding circulating protein levels, were enhanced by SPI and WPH.

**Conclusions:**

The identification of transcripts co- or differentially-regulated by SPI and WPH diets suggests common as well as unique anti-tumorigenesis mechanisms of action which may involve growth factor, neuroendocrine and immune system genes. SPI and WPH induction of somatostatin, a known anti-proliferative agent for colon cancer cells, would inhibit tumorigenesis.

## Background

Colorectal cancer (CRC) is the third most common cancer and the third leading cause of cancer-related mortality in the U.S. [1,2]. Estimated new cases of colon cancer were 79,650 for men and 73,530 for women in 2004 [1]; approximately $6.3 billion is spent in the United States each year on treatment of CRC [2]. Accumulating evidence suggests that diet is an important environmental factor in the etiology of CRC. High consumption of red meats, animal fats, chocolate, alcohol and refined cereals are linked to higher incidence of these cancers in Western societies [3-5], whereas protective effects of fruits, vegetables and whole grains have been suggested [5].

Soy foods and soybean constituents have received considerable attention for their potential role in reducing cancer risk [6,7]. Our laboratories reported the protective effects of lifetime ingestion of soy protein isolate (SPI) on azoxymethane (AOM)-induced colon cancer in rats [8]. Similarly, the effect of whey protein hydrolysate (WPH) in the diet to reduce colon tumor incidence has been reported by us and others [9-11]. Several hypotheses have been proposed to account for soy and whey protein-induced anti-tumorigenesis. For example, soy isoflavones have been proposed to play a key role in soy's anti-cancer functions [12]. Yanagihara *et al*., among others, reported that genistein inhibits colon cancer cell proliferation and stimulates apoptosis in vitro [13-15]. However, subcutaneous administration of genistein to mice did not confirm these *in vitro *effects [16]. Holly *et al*. reported that soy sphingolipids inhibit colonic cell proliferation, and suggested that this may partially account for its anticancer benefits [17]. Other reports indicate that soy diets inhibit tumorigenesis by regulating the synthesis or activities of specific proteins. For example, Rowlands *et al*. reported that dietary soy and whey proteins down-regulate expression of liver and mammary gland phase I enzymes involved in carcinogen activation [18]. Elevated activities of phase II detoxification enzymes were reported in soy-fed rats [19,20]. Such dietary effects may result in lower tissue concentrations of activated carcinogen. The anticancer properties of whey proteins have been ascribed to their ability to elevate cellular levels of the antioxidant glutathione [21,22]. Moreover, the whey protein, α-lactalbumin, inhibits proliferation of mammary epithelial cells in vitro [11]. The anticancer properties of whey may also relate to its immune system-enhancing actions [23].

Despite extensive research, there is no consensus for anti-cancer mechanism(s) of soy and whey, which will undoubtedly involve multiple interrelated processes, pathways and many components. Many of the same molecular and biochemical changes underlying human colon cancer are observed in the azoxymethane (AOM)-induced rat colon cancer model [24]. Moreover, previous studies suggest a different molecular etiology for tumors of the proximal and distal colon in this model and in human colon [24,25]. Differential dietary effects on proximal vs. distal colon DNA damage were noted [26] and Westernization of the human diet is thought to have favored a shift of tumors from distal to more proximal locations [27]. Thus, region-specific localization of dietary effects on colon tumorigenesis is an important factor to consider in any molecular analysis of CRC. Here, we use Affymetrix high-density oligonucleotide microarrays to determine the expression profiles of non-tumor (i.e., normal) tissue in proximal colons (PC) of rats, subjected to lifetime diets containing casein (CAS, control diet), soy protein isolate (SPI), or whey protein hydrolysate (WPH) and which were administered AOM to induce tumors. We hypothesized that genes whose expression contributes to anti-tumorigenesis would be regulated in parallel by SPI and WPH; in addition, changes unique to each diet might also be apparent.

## Results

### Validation of the microarray approach

Quality control steps ensured that the RNA used for microarray and real-time RT-PCR analysis was of high quality. These steps included evaluation of the RNA with the RNA 6000 Nano Assay and assessment of the cRNA hybridization to GeneChips by comparison of data obtained for probe sets representative of 5' and 3' ends of control genes. All RNA samples had an A260/280 absorbance ratio between 1.9 and 2.1. The ratio of 28S to 18S rRNA was very close to 2 on RNA electropherograms, and signal ratios below 3 were noted for 3' vs. 5' probe sets for β-actin and glyceraldehyde-3-phosphate dehydrogenase (per Affymetrix user guidelines) after hybridization.

**Figure 1:**
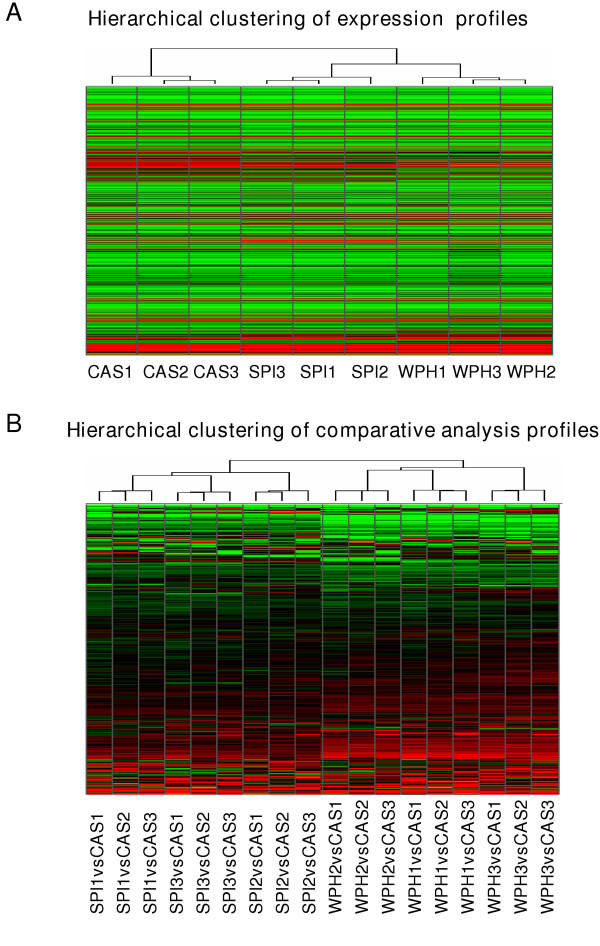
Hierarchical clustering of proximal colon gene expression profiles. **A. **Clustering of nine PC global gene expression profiles (8799 genes); n = 3 profiles each for CAS, SPI and WPH. Each cell represents the expression level of an individual gene in each sample (green = low expression, black = middle expression, red = high expression). The dendrogram reflects the extent of relatedness of different profiles; the shorter branch-point of the SPI and WPH trees indicates the greater similarity between these profiles. **B. **Clustering of 18 global comparative expression profiles including 9 of SPI vs. CAS and 9 of WPH vs. CAS profiles. Each row in the heat map represents the relative expression level of a given gene across all comparisons (red = up regulated, black = unchanged, green = down regulated).

**Figure 2:**
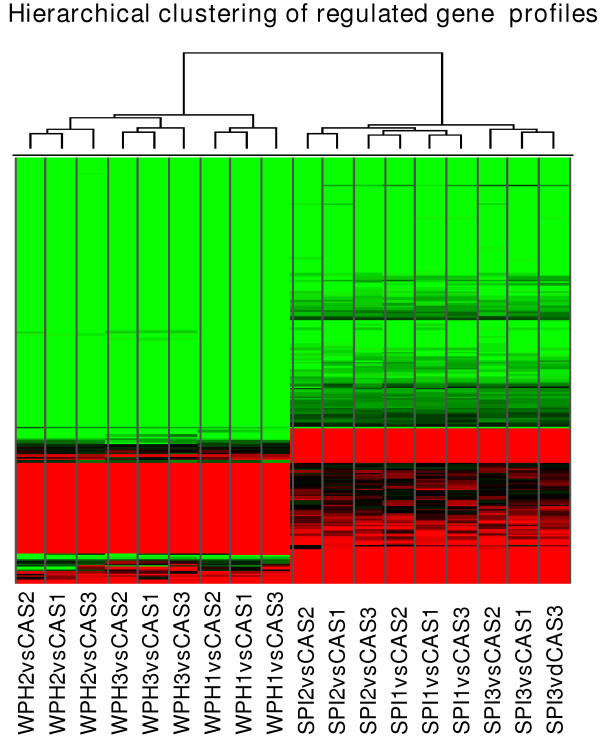
Hierarchical clustering of 211 differentially expressed genes in either SPI or WPH. The differential expression data are taken only from the pairwise comparison analysis, with CAS profiles used as baseline. Each cell in the heat map represents the relative expression level of a given gene in an individual comparison analysis (red = up regulated, black = unchanged, green = down regulated). The dendrogram reflects the relatedness of different profiles.

Total false change rates (TFC) were determined following Affymetrix-recommended guidelines [28], except that the inter-chip comparisons used cRNA targets made in parallel starting from the same RNA pool. Inter-chip variability, measured as TFC%, was 0.25% – 0.6% and well below the suggested 2% cutoff (Table 1). These values confirmed the fidelity and reproducibility of the microarray procedures used. Unsupervised nearest-neighbor hierarchical clustering identified differences in proximal colon gene expression profiles of CAS, SPI and WPH groups (Figs. 1 and 2), indicating that the type of dietary protein has a major effect on gene expression in normal proximal colon tissue of AOM-treated rats. Interestingly, the overall gene expression profiles for SPI and WPH groups were more similar to each other than each was to the CAS group (Fig. 1A).

**Table 1:** Inter-chip variability

Diet group	Number of arrays	TFC (%)*
CAS	3	0.252 ± 0.138
WPH	3	0.369 ± 0.025
SPI	3	0.570 ± 0.165

*TFC (Total false change) = false change rate (decreased category) + false change rate (increased category), as described in ref. 28; TFC reported as mean ± SEM, TFC should be no more than 2% (Affymetrix).

### Differentially expressed genes

Multiple filtering criteria were applied to the microarray data set so as to identify differentially expressed colon transcripts in rats fed SPI, WPH or CAS; results are reported only for transcripts that passed all three analytical filters used: DMT t-test, SAM and pair-wise comparison survival methods. Among the 8799 genes and ESTs examined with the rat U34A array, we identified 31 induced and 49 repressed genes in proximal colons of SPI-fed rats, whereas 44 induced and 119 repressed genes were detected in WPH-fed rats (Tables 2, 3, 4, 5). Interestingly, more down- than up-regulated genes were noted for both SPI and WPH. Additionally, 37 genes were co-repressed, whereas only two were co-induced by SPI and WPH (Table 6). More than 90% of identified genes in WPH and SPI animals showed the same direction of change relative to CAS. This is visually apparent in the hierarchical clustering output (Fig. 2).

**Table 2:** Down-regulated genes in rats fed with WPH diet*

Category and Gene Name	Probe Set GB Accession No.	Fold Change	P value
***Cell adhesion***			
Embigin	AJ009698	-6.57	0
Cadherin 17	L46874	-4.8	0.036
Cadherin	X78997	-3.36	0.004
Protein tyrosine phosphatase	M60103	-2.64	0.004
Cytokeratin-8	S76054	-2.71	0
Trans-Golgi network integral membrane protein TGN38	X53565	-4.92	0.012
Tumor-associated calcium signal transducer 1	AJ001044	-9.37	0.001
Claudin-3	AJ011656	-7.55	0.02
Claudin-9	AJ011811	-5.12	0
***Cell cycle/growth control***			
Mapk6	M64301	-2.61	0.003
Epithelial membrane protein 1	Z54212	-4.67	0.015
Glucagon	K02813	-7.73	0.005
Peptide tyrosine-tyrosine (YY)	M17523	-4.56	0.001
Src related tyrosine kinase	U09583	-3.31	0.033
FGF receptor activating protein	U57715	-4.25	0.002
Cyclin D1	D14014	-1.97	0.001
Neu oncogene	X03362	-2.61	0.017
***Defense/immunity protein***			
Seminal vesicle secretion protein iv	J00791	-5.35	0.001
Putative cell surface antigen	U89744	-5.22	0.008
Decay accelerating factor GPI	AF039583	-6.12	0
Beta defensin-1	AF093536	-26.78	0.001
***Detoxification/antioxidation***			
Glutathione S-transferase	J02810	-5.17	0
Glutathione S-transferase Yb	X04229	-9.33	0
	J03914	-2.43	0.002
Glutathione S-transferase, alpha 1	K01932	-3.07	0.002
Glutathione transferase, subunit 8	X62660	-6.42	0.001
Glutathione S-transferase Yc1	S72505	-3.69	0.004
Glutathione S-transferase Yc2	S72506	-21.38	0.008
N-acetyltransferase 1	U01348	-4.64	0.003
Cytochrome P450CMF1b	J02869	-8.23	0.001
Cytochrome P450 4F4	U39206	-6.43	0.004
Cytochrome P450 monooxygenase	U39943	-2.82	0.011
Cytochrome P450 pseudogene	U40004	-2.87	0
Cytochrome P450 3A9	U46118	-6.91	0
Cytochrome P450IVF	M94548	-5.78	0.002
Cytochrome P450, subfamily 51	U17697	-2.07	0.005
Alcohol dehydrogenase	M15327	-2.06	0.025
Aldehyde dehydrogenase	M23995	-10.56	0.035
	AF001898	-2.72	0.004
D-amino-acid oxidase	AB003400	-13.69	0
3-methylcholanthrene-inducible UDP-glucuronosyltransferase	S56937	-9	0
UDP-glucuronosyltransferase	D38062	-3.17	0.005
	D38065	-3.29	0.002
UDP glycosyltransferase 1	D83796	-6.87	0
	J02612	-6.58	0
	J05132	-4.03	0
***Metabolism***			
Meprin 1 alpha	S43408	-3.82	0.014
Brain serine protease bsp1	AJ005641	-4.42	0.007
Cystathionine gamma-lyase	D17370	-3.05	0.002
Cathepsin S	L03201	-2.62	0
Meprin beta-subunit	M88601	-5	0.004
Disintegrin and metalloprotease domain 7	X66140	-11.91	0
Fucosyltransferase 1	AB006137	-4.96	0.001
Fucosyltransferase 2	AB006138	-7.97	0.017
UDP-glucose:ceramide glycosyltransferase	AF047707	-2.86	0.007
Type II Hexokinase	D26393	-2.7	0.001
Hexokinase II	S56464	-4.45	0.007
CDP-diacylglycerol synthase	AB009999	-4.66	0
Carboxylesterase precursor	AB010635	-5.29	0.002
Fatty acid Coenzyme A ligase	AB012933	-2.5	0.041
3beta-HSD	L17138	-3.27	0
11-beta-hydroxylsteroid dehydrogenase type 2	U22424	-3	0.001
Peroxiredoxin 6	AF014009	-3.55	0.01
Platelet phospholipase A2	X51529	-3.25	0.001
***Ligand binding/carrier***			
Carnitine transporter	AB017260	-3.95	0.005
Chloride channel (ClC-2)	AF005720	-5.69	0.002
Putative potassium channel	AF022819	-4.84	0
Mitochondrial dicarboxylate carrier	AJ223355	-3.55	0.009
Aquaporin 3	D17695	-7.83	0
Na_H_Exchanger	L11236	-9.81	0.003
Angiotensin/vasopressin receptor (AII/AVP)	M85183	-3.3	0.002
H+, K+-ATPase	M90398	-13.87	0
Intestinal fatty acid binding protein	K01180	-7.29	0.001
Apolipoprotein A-I precursor	M00001	-3.45	0.023
Apolipoprotein A-I	J02597	-2.47	0.003
Sodium-hydrogen exchange protein-isoform 3	M85300	-7.36	0.004
Liver fatty acid binding protein	V01235	-2.62	0
Sodium transporter	X59677	-3.8	0
Cation transporter	X78855	-3.62	0.003
ATP-binding cassette	AB010467	-3.89	0.004
Methionine adenosyltransferase II, alpha	J05571	-2.91	0.007
Phenylalanine hydroxylase	M12337	-7.43	0
Carbonic anhydrase IV	S68245	-4.28	0.011
***Signal transduction***			
B7 antigen	X76697	-170.95	0.002
CD24 antigen	U49062	-3.08	0
Chemokine CX3C	AF030358	-5.04	0.011
Itmap1	AF022147	-7.5	0.001
HCNP	E05646	-2.5	0.001
Brain glucose-transporter protein	M13979	-2.97	0.019
Protein kinase C delta	M18330	-2.48	0.002
Guanylate cyclase 2C	M55636	-4.58	0.003
A2b-adenosine receptor	M91466	-2.8	0.04
Guanylate cyclase activator 2A	M95493	-4.18	0.005
Phospholipase C beta-3	M99567	-2.57	0.018
Tm4sf3	Y13275	-3.33	0
Phospholipase D	AB000778	-2.71	0.009
BEM-2	D45413	-6.41	0.015
Sgk	L01624	-3.93	0
***Stress response/apoptosis***			
Prostaglandin D synthetase	J04488	-43.11	0.009
GTP cyclohydrolase I	M58364	-3.26	0.014
***Structure proteins***			
Chromogranin B (Chgb)	AF019974	-2.56	0.005
Intestinal mucin	M76740	-5.09	0.002
Muc3	U76551	-11.07	0.006
Mucin-like protein	M81920	-11.97	0.001
Myosin 5B	U60416	-3.94	0
Keratin 18	X81448	-3.23	0.004
Keratin 19	X81449	-2.69	0.001
ZG-16p protein	Z30584	-4.43	0.002
Plasmolipin	Z49858	-7.2	0
Cytokeratin 21	M63665	-4.96	0
Syndecan	S61865	-3.3	0.006
Claudin 3	M74067	-6.68	0.01
***Transcription factor/regulator***			
Hepatocyte nuclear factor 3 gamma	AB017044	-6.96	0
Apolipoprotein B mRNA editing protein	L07114	-2.34	0
DNA-binding inhibitor	L23148	-4.1	0.01
Kruppel-like factor 4 (gut)	L26292	-3.08	0.017
***Others***			
Prolactin receptor	M74152	-3.26	0.014
LOC286964	U89280	-2.96	0.003
Ckmt1	X59737mRNA	-2.65	0.025
Arginase II	U90887	-23.69	0
Deleted in malignant brain tumors 1	U32681	-3.47	0.002
3' end GAA-triplet repeat	L13025	-2.73	0.001
Polymeric immunoglobulin receptor	L13235	-2.93	0.004

*Changes in gene expression were determined by t-test (DMT), comparative analysis (MAS 5.0), and SAM (Stanford). Gene expression profiles from CAS animals were used as control. *P *value and fold change are based on DMT analysis; whereas final genes listed met all of the analytical criteria as described in Methods.

**Table 3:** Up-regulated genes in rats fed with WPH diet*

Category and Gene Name	Probe Set GB Accession No.	Fold Change	P value
***Cell adhesion***			
Fibronectin	X05834	2.3	0
EGF-containing fibulin-like extracellular matrix protein 1	D89730	2.17	0.004
**Cell cycle/growth control**			
Somatostatin	M25890	2.72	0.001
Somatostatin-14	K02248	3.87	0.009
APEG-1	U57097	3.24	0.002
***Defense/immunity protein***			
IgG gamma heavy chain	M28670	2.21	0.009
T-cell receptor beta chain	X14319	2.14	0
Adipsin	M92059	3.21	0
***Ligand binding/carrier***			
Angiotensin receptor	M86912	2.75	0.017
Calretinin	X66974	2.52	0.005
Purkinje cell protein 4	M24852	3.06	0.001
Secretogranin III	U02983	2.77	0.005
Secretogranin II	M93669	2.84	0.001
Aquaporin 1	X67948	3.4	0.008
Cacna2d1	M86621	2.84	0
Retinol-binding protein	M10934	2.17	0.018
***Metabolism***			
Lipoprotein lipase	L03294	2.72	0
Ubiquitin carboxyl-terminal hydrolase	D10699	3	0.003
***Signal transduction***			
Thy-1 protein	X02002	2.89	0.002
CD3 gamma-chain	S79711	3.28	0.002
Synapsin	M27925	3.94	0.001
Alpha-actinin-2 associated LIM protein	AF002281	2.74	0.009
RESP18	L25633	2.74	0.033
T3 delta protein	X53430	2.75	0.003
Protein phosphatase inhibitor-1	J05592t	2.6	0.009
CART protein	U10071	2.16	0.001
Neuroendrocrine protein	M63901	3.7	0.006
Protein kinase C-binding protein Zeta1	U63740	3.14	0.003
cannabinoid receptor 1	X55812	2.17	0.002
Guanylyl cyclase A	J05677	3.18	0.007
Tachykinin 1	X56306	2.36	0.036
Protein tyrosine phosphatase	L19180	2.47	0.041
Argininosuccinate synthetase	X12459	4.69	0.004
***Stress response/apoptosis***			
Small inducible cytokine	Y08358	3.35	0.029
***Structure proteins***			
Fast myosin alkali light chain	L00088	4.52	0.03
Light molecular-weight neurofilament	AF031880	2.41	0
Neurofilament protein middle	Z12152	2.97	0.006
Alpha-tubulin	V01227	2.25	0
Peripherin	AF031878	2.82	0.007
***Transcription factor/regulator***			
snRNP	M29293	2.11	0.004
snRNP-associated polypeptide	X73411	3.33	0.002
***Others***			
C1-13 gene product	X52817	3.17	0
ND5, ND6	S46798	2.31	0.015
Sensory neuron synuclein	X86789	2.84	0

*Changes in gene expression were determined by t-test (DMT), comparative analysis (MAS 5.0), and SAM (Stanford). Gene expression profiles from CAS animals were used as control. *P *value and fold change are based on the DMT analysis; whereas listed genes met all of the analytical criteria as described in Methods.

**Table 4:** Down-regulated genes in rats fed with SPI diet*

Category and Gene Name	Probe Set GB Accession No.	Fold Change	P value
***Cell adhesion***			
Embigin	AJ009698	-5.13	0.001
***Cell Cycle/growth control***			
FGF receptor activating protein 1	U57715	-5.59	0.002
BEST5 protein	Y07704	-2.37	0.003
Peptide tyrosine-tyrosine (YY)	M17523	-3.91	0.002
Glucagon gene	K02813	-6.58	0.002
Epithelial membrane protein-1	Z54212	-3.47	0.017
Neu oncogene	X03362	-1.58	0.05
***Defense/immunity protein***			
Beta defensin-1	AF068860	-42.16	0.001
	AF093536	-10.2	0
**Detoxification/antioxidation**			
Glutathione S-transferase	J02810	-7.14	0
Glutathione S-transferase Yb	X04229	-11.71	0.001
Glutathione S-transferase, alpha 1	K01932	-4.18	0.004
Glutathione S-transferase Yc1	S72505	-5.23	0.001
Glutathione S-transferase Yc2	S72506	-5.27	0.012
	S82820	-3.45	0.006
Cytochrome P450 4F4 (CYP4F4)	U39206	-6.52	0.002
Cytochrome P450CMF1b	J02869	-4.12	0.002
Cytochrome P450 (CYP4F1)	M94548	-2.88	0.002
1-Cys peroxiredoxin	Y17295	-2.55	0.002
Metallothionein	M11794	-2.92	0.006
D-amino-acid oxidase	AB003400	-5.42	0
Peroxiredoxin 6	AF014009	-3.07	0.008
Phenylalanine hydroxylase	M12337	-10.99	0.001
***Metabolism***			
Dipeptidase	L07315	-3.08	0.001
Meprin beta-subunit	M88601	-3.27	0.001
Disintegrin and metalloprotease domain 7	X66140	-14.03	0
***Ligand binding/carrier***			
Carnitine transporter	AB017260	-3.81	0.003
Chloride channel (ClC-2)	AF005720	-3.26	0.001
Putative potassium channel	AF022819	-2.69	0.001
Mitochondrial dicarboxylate carrier	AJ223355	-2.54	0.01
Aquaporin 3	D17695	-4.13	0
Intestinal fatty acid binding protein	K01180	-4.43	0.005
Na_H_Exchanger	L11236	-4.47	0.002
H+, K+-ATPase	M90398	-2.52	0.001
Carbonic anhydrase IV	S68245	-4.28	0.005
Sodium transporter	X59677	-3.4	0
Phosphatidylethanolamine binding protein	X75253	-2.69	0
***Signal transduction***			
B7 antigen	X76697	-170.95	0.002
HCNP	E05646	-3.38	0
Itmap1	AF022147	-7.97	0.005
Guanylate cyclase activator 2A	M95493	-3.28	0.006
Sgk	L01624	-2.76	0
***Stress response/apoptosis***			
Prostaglandin D synthetase	J04488	-45.8	0.01
***Structure proteins***			
Muc3	U76551	-3.56	0.01
Intestinal mucin	M76740	-3.31	0.006
Mucin-like protein	M81920	-3	0
Plasmolipin	Z49858	-2.92	0.003
***Transcription factor/regulator***			
Testis specific X-linked gene	X99797	-6.91	0.003
***Others***			
Arginase II	U90887	-3.22	0
3-phosphoglycerate dehydrogenase	X97772	-4.15	0.017
Aldehyde dehydrogenase family 1	AF001898	-3.93	0.004

*Changes in gene expression were determined by t-test (DMT), comparative analysis (MAS 5.0), and SAM (Stanford). Gene expression profiles from CAS animals were used as control. *P *value and fold-change are based on the DMT analysis; whereas genes listed above met all of the analytical criteria as described in Methods.

**Table 5:** Up-regulated genes in rats fed with SPI diet*

Category and Gene Name	Probe Set GB Accession No.	Fold Change	P value
***Cell adhesion***			
Collagen alpha1 type I	Z78279	2.49	0
Secreted phosphoprotein 1	M14656	111.39	0.006
Matrix metalloproteinase 13	M60616	24.34	0.002
Regenerating islet	M62930	193.08	0.011
***Defense/immunity protein***			
Ig gamma-2a chain	L22654	115.17	0.001
Ig gamma heavy chain	M28670	3.22	0
Ig germline kappa-chain C-region	M18528	2.48	0.038
Ig light-chain	U39609	2.63	0.021
Fc-gamma	M32062	4.72	0.017
***Detoxification***			
Glutathione S-transferase 1	J03752	2.86	0
Glutathione-S-transferase,alpha type2	K00136	2.56	0.009
UDP glucuronosyltransferase	D38066	2.83	0.014
***Metabolism***			
Matrix metalloproteinase 7	L24374	3.63	0.02
lysozyme	rc_AA892775	2.77	0
Matrix metalloproteinase 12	X98517	11.8	0.013
Mitochondrial carbamyl phosphate synthetase I	M12335	59.25	0.001
Aldolase B, exon 9	X02291	8.7	0.01
Aldolase B, exon 2	X02284	2.71	0.001
***Signal transduction***			
MHC class II antigen RT1.B-1 beta-chain	X56596	2.55	0.001
CD3 gamma-chain	S79711	4.51	0.001
***Ligand binding/carrier***			
Intracellular calcium-binding protein	L18948	28.29	0.014
Retinol binding protein II	M13949	5.11	0.001
Apolipoprotein B	M27440	6.47	0.024
Apolipoprotein A-I	J02597	2.49	0.004
Iron ion transporter	AF008439	18.78	0.008
***Stress response/apoptosis***			
Heme oxygenase	J02722	9.66	0.002
JE product	X17053	3.52	0.001
Pancreatitis-associated protein	M98049	68.39	0.004
Pancreatitis associated protein III	L20869	15.35	0
Reg protein	E01983	30.25	0.001
**Others**			
Histamine N-tele-methyltransferase	S82579	6.17	0.04

*Changes in gene expression were determined by t-test (DMT), comparative analysis (MAS 5.0), and SAM (Stanford). Gene expression profiles from CAS animals were used as control. *P *value and fold-change are based on the DMT analysis; whereas final listed genes met all of the analytical criteria as described in the Methods.

**Table 6:** Genes co-regulated with WPH and SPI diet*

Category and Gene Name	Probe Set GB Accession No.	Fold Change in WPH	P value	Fold Change in SPI	P value
***Down-regulated genes***					
Embigin	AJ009698	-6.57	0	-5.13	0.001
Epithelial membrane protein 1	Z54212	-4.67	0.015	-3.47	0.017
Glucagon	K02813	-7.73	0.005	-6.58	0.002
Peptide tyrosine-tyrosine (YY)	M17523	-4.56	0.001	-3.91	0.002
FGF receptor activating protein	U57715	-4.25	0.002	-5.59	0.002
Neu oncogene	X03362	-2.61	0.017	-1.58	0.05
CD52 antigen	X76697	-170.95	0.002	-170.95	0.002
Beta defensin-1	AF068860	-54.48	0.001	-42.16	0.001
Glutathione S-transferase	J02810	-5.17	0	-7.14	0
Glutathione S-transferase Yb	X04229	-9.33	0	-11.71	0.001
Glutathione S-transferase, alpha 1	K01932	-3.07	0.002	-4.18	0.004
Glutathione S-transferase Yc1	S72505	-3.69	0.004	-5.23	0.001
Glutathione S-transferase Yc2	S72506	-21.38	0.008	-5.27	0.012
Cytochrome P450CMF1b	J02869	-8.23	0.001	-4.12	0.002
Cytochrome P450 4F4	U39206	-6.43	0.004	-6.52	0.002
Cytochrome P450IVF	M94548	-5.78	0.002	-2.88	0.002
D-amino-acid oxidase	AB003400	-13.69	0	-5.42	0
Meprin beta-subunit	M88601	-5	0.004	-3.27	0.001
Disintegrin and metalloprotease domain 7	X66140	-11.91	0	-14.03	0
Carnitine transporter	AB017260	-3.95	0.005	-3.81	0.003
Chloride channel (ClC-2)	AF005720	-5.69	0.002	-3.26	0.001
Putative potassium channel	AF022819	-4.84	0	-2.69	0.001
Mitochondrial dicarboxylate carrier	AJ223355	-3.55	0.009	-2.54	0.01
Aquaporin 3	D17695	-7.83	0	-4.13	0
Na_H_Exchanger	L11236	-9.81	0.003	-4.47	0.002
H+, K+-ATPase	M90398	-13.87	0	-2.52	0.001
Fatty acid binding protein 1	K01180	-7.29	0.001	-4.43	0.005
Sodium transporter	X59677	-3.8	0	-3.4	0
Carbonic anhydrase IV	S68245	-4.28	0.011	-4.28	0.005
Itmap1	AF022147	-7.5	0.001	-7.97	0.005
HCNP	E05646	-2.5	0.001	-3.38	0
Guanylate cyclase activator 2A	M95493	-4.18	0.005	-3.28	0.006
Sgk	L01624	-3.93	0	-2.76	0
Prostaglandin D synthetase	J04488	-43.11	0.009	-45.8	0.01
Mucin 3	M76740	-5.09	0.002	-3.31	0.006
Mucin-like protein	M81920	-11.97	0.001	-3	0
Plasmolipin	Z49858	-7.2	0	-2.92	0.003
***Up-regulated genes***					
Ig gamma heavy chain	M28670	2.21	0.009	3.22	0
CD3 gamma-chain	S79711	3.28	0.002	4.51	0.001

*Changes in gene expression were determined by t-test (DMT), comparative analysis (MAS 5.0), and SAM (Stanford). Gene expression profiles from CAS animals were used as control. *P *value and fold change are based on the DMT analysis; whereas final listed genes met all of the analytical criteria described in Methods.

### Gene expression: effects of WPH

As based on Gene Ontology (GO) annotations, the 44 up-regulated and 119 down-regulated genes of the WPH group belong to multiple functional categories including cell adhesion (n = 10), cell cycle and growth control (n = 10), detoxification (n = 17), defense and immunity (n = 7), signal transduction (n = 29), transcriptional regulation (n = 6), metabolism (n = 19), ligands and carriers (n = 27), cell death (n = 3), structural proteins (n = 16), and others (Tables 2 &3). The fold change for up-regulated genes ranged between 2.1 [small nuclear ribonucleoparticle-associated protein (snRNP)] to 4.7 (argininosuccinate synthetase), whereas down-regulated genes exhibited fold changes between 2.0 (cyclin D1) and 171 (CD52 antigen).

Lifetime ingestion of WPH affected the expression of xenobiotic metabolism-related enzymes including several of the cytochrome P450s and glutathione S-transferases, alcohol dehydrogenase (ADH), and UDP-glucuronosyltransferase. Cytochrome P450 enzymes and ADH are considered to play key roles in activation of the proximate carcinogen from AOM [29]. Down-regulation of expression of Phase I detoxification enzymes by WPH might therefore diminish AOM-induced DNA adducts and genomic instability. Consistent with results from a study in which whey proteins inhibited cell proliferation *in vitro *[11], lifetime feeding of WPH was associated with changes in expression of genes involved in cell cycle control and proliferation; cyclin D1, neu oncogene, mapk6, glucagon, and peptide YY (PYY) genes were down-regulated, whereas the expression of somatostatin, a growth-inhibitory peptide was induced. WPH altered expression of genes involved in cellular defense. Induced genes included Ig gamma heavy chain, adipsin, and T-cell receptor beta chain, whereas expression of the antibacterial peptide beta defensin-1 and seminal vesicle secretion protein IV (SVS IV) were down-regulated. About 20% of WPH-affected genes are involved in cell signaling; these include guanylate cyclase 2C, protein kinase C delta, and synapsin. Additionally, genes encoding ligands or membrane channels [i.e., chloride channel, intestinal fatty acid binding protein (I-FABP), apoliprotein A-I (Apo-AI), Na+, K+-ATPase, and sodium transporter] were down-regulated by WPH, whereas calretinin and retinol binding protein (RBP) levels were increased.

### Gene expression: effects of SPI

Colon genes, whose mRNA expression was affected by ingestion of SPI, fell into multiple functional categories including cell adhesion (n = 4), cell cycle and growth control (n = 6), detoxification (n = 18), defense and immunity (n = 6), signal transduction (n = 4), transcriptional regulation (n = 1), metabolism (n = 8), ligands and carrier proteins (n = 17), cell death proteins (n = 5), and structural proteins (n = 3) (Tables 4 &5).

Relative abundance of numerous transcripts was changed in the same direction by WPH and SPI (Fig. 2). However, some exceptions were noted. For example, mRNA encoding Apo-AI was down-regulated by WPH, but elevated by SPI. Apo-AI is the major determinant of the capacity of HDL particles to promote cholesterol efflux and this protein is associated with the inhibition of atherosclerosis [30]. However, the impact of differential response of Apo-AI to WPH and SPI on anti-tumorigenesis is unknown.

### Confirmation of differential gene expression

**Figure 3:**
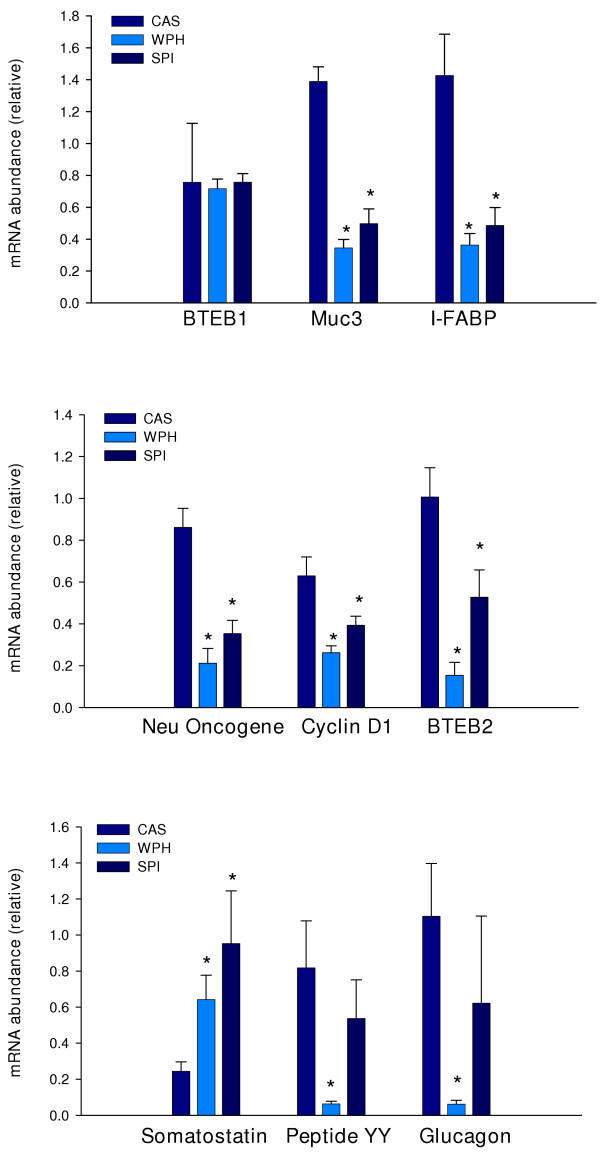
Quantitative real-time RT-PCR verification of microarray results. RNA used for real-time RT-PCRs was from the same animals (n = 7 per diet group) whose RNAs comprised the pools for microarray analysis. Values are mean ± SEM and were analyzed by one-way ANOVA, *P < 0.05, SPI or WPH vs. CAS.

We performed quantitative real-time RT-PCR on selected genes to confirm the microarray results. Based upon known associations with cell proliferation or differentiation, 14 genes were chosen for further study. Included in this group was BTEB2; this gene was not present on the microarrays but was included in RT-PCR analysis due to its significant expression in intestine and involvement in cell proliferation [see discussion]. As shown in Figure 3, eight genes were confirmed to be differentially expressed: these included the gastrointestinal hormone genes PYY (12.9-fold down-regulated in WPH fed rats; P = 0.004), glucagon (17.8-fold down-regulated in WPH fed rats; P = 0.005), and somatostatin (3.92-, 2.65-fold up-regulated in SPI and WPH fed rats; P = 0.05, P = 0.025, respectively); cyclin D1 (1.6-, 2.4-fold down-regulated in SPI and WPH fed rats; P = 0.033, P = 0.001, respectively); BTEB2 (1.9-, 6.7-fold down-regulated in SPI and WPH fed rats; P = 0.024, P < 0.001, respectively); c-neu proto-oncogene (2.5-, 4.1-fold down-regulated in SPI and WPH fed rats; P < 0.001, P < 0.001, respectively); the colonocyte differentiation marker I-FABP (2.9-, 4.0-fold down-regulated in SPI and WPH fed rats; P = 0.023, P = 0.01, respectively); and the mucin, MUC3 (2.78-, 4.05-fold down-regulated in SPI and WPH fed rats; P < 0.001, P < 0.001, respectively). Differential expression of five other genes was not confirmed statistically, due to individual animal variation in the transcript levels; however, the mean-fold changes for mRNA abundance were greater than two and in agreement with the corresponding microarray results for these genes. Only one of the selected genes – retinol binding protein (RBP), failed to exhibit greater than a 2-fold change (in the predicted direction) at the mRNA level by real-time RT-PCR.

### Serum somatostatin (Sst)

**Figure 4:**
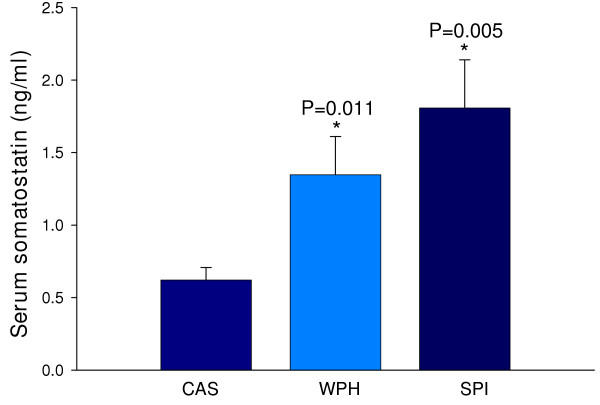
Diet effects on serum Sst concentration. Values are mean ± SEM. One-way ANOVA. *P < 0.05, SPI or WPH vs. CAS.

As shown in Fig. 4, circulating Sst concentration was significantly higher in rats fed WPH and SPI. Colonic Sst protein content in colon homogenates was below the limit of detection of the assay used (data not shown).

## Discussion

The type of dietary protein(s) can markedly affect the onset and/or progression of CRC [31]. Epidemiological and animal studies have found that dietary soy and whey proteins decrease the incidence of certain tumors, including those of the colon and rectum [6,7,32-35]. Using the AOM-treated male Sprague Dawley rat model, we previously found that lifetime feeding of SPI led to a ~ 76% lower incidence of AOM-induced colon tumors compared to rats lifetime-fed CAS [8]. Additionally, in the same studies, a ~ 46% lower incidence of colon tumors was found in WPH-fed compared to CAS-fed rats [9]. The molecular mechanism(s) by which these dietary proteins reduce the incidence of chemically-induced colon tumors is unclear, although several mechanisms and putative bio-active factors have been proposed [11-24]. The present study has now identified genes that are differentially expressed as a function of these diets and which serve to highlight potential pathways for dietary protection from carcinogenesis.

**Figure 5:**
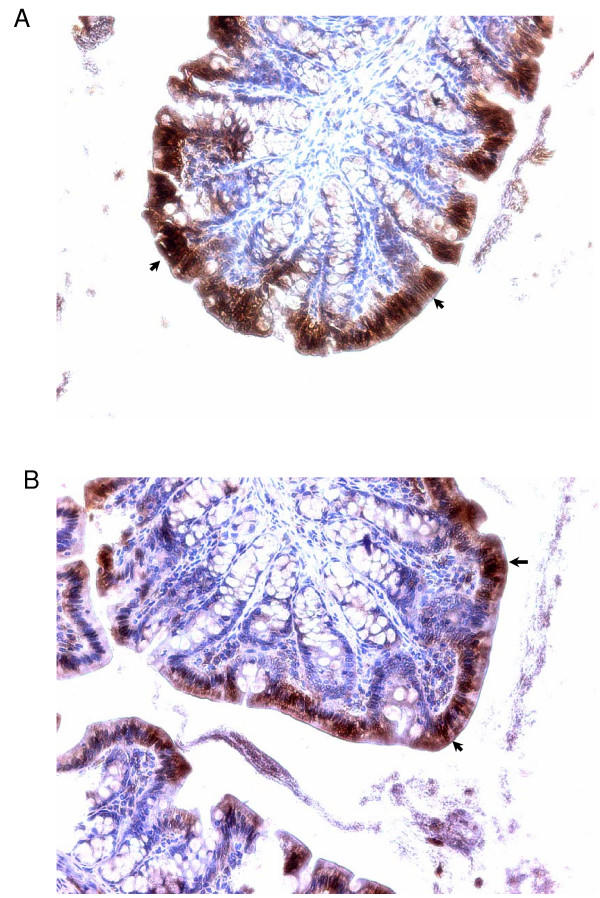
Immuno-histochemistry for I-FABP in colons from AOM-treated rats. Panels A and B are sections from CAS and WPH-fed animals, respectively. Arrows point to the strong areas of staining for I-FABP in the inter-cryptal surface epithelium (overall intensity of staining is greater for CAS than for WPH).

The ability to simultaneously analyze a large number of different mRNAs makes microarrays very appealing for identifying genes and gene families whose expression is altered by diet [36,37]. We focused on the 'normal' colon tissue since we are interested in genes that are differentially regulated by diet and which act in anti-oncogenic fashion in pre-cancerous tissues. We limited our analysis to the proximal colon since several studies have suggested that the molecular etiology of proximal and distal colon tumors differs [25,26] and proximal colon tumors have become more prevalent with Westernization of the diet and aging of the population [27]. We chose to include colonic smooth muscle with the mucosa since: a) the former tissue layer interacts with the latter to influence its growth and function, and b) we could monitor all colonic genes affected by diet. However, one potential caveat to this strategy is the 'dilution' effect that may have been imposed on the more rare mucosal transcripts. Another caveat is that no information is obtained regarding where the differentially expressed transcripts occur. In this regard, however, we have confirmed by immuno-histochemistry that I-FABP is expressed predominantly in the inter-cryptal surface epithelium of colons from AOM-treated rats (Fig. 5). Our study used a sample size of three (per diet group) which balanced the costs for the experimental reagents with the minimum number required for statistical analysis. The quantitative PCR analyses provided confirmation that the filtering strategies used yielded bona-fide differentially expressed transcripts.

Only two transcripts were induced by both SPI and WPH; whereas 37 transcripts were repressed by both SPI and WPH. This suggests that the cancer-protective actions of the two diets are generally associated with repression of colonic genes that facilitate tumorigenesis. An alternative explanation is that CAS induces genes that facilitate colon cancer development when compared to SPI and WPH. It is also likely that SPI and WPH diets act in unique ways to inhibit tumorigenesis. Irregardless, our results indicate that the nature of the dietary protein can profoundly affect colon gene expression profiles. Thus, gene expression profiling studies of colons should account for potential confounding effects of diet.

Dietary factors in SPI or WPH inhibit cell proliferation and induce apoptosis among other biological actions [11,13]. In the present study, we identified cyclin D1 gene and the neu proto-oncogene as being repressed in proximal colon by SPI and WPH. Cyclin D1 is a key regulator of cell cycle progression [38], and a target of β-catenin, a protein whose abnormal accumulation in the nucleus is strongly linked to the development of multiple tumor types, including those of the colon [39]. Aberrantly increased expression of cyclin D1 in colon epithelial cells contributes to their abnormal proliferation and tumorigenicity [40,41]. Similarly, the oncogenic and cellular growth-promoting activities of the HER-2/neu proto-oncogene are well known [42]. HER-2/neu, a tyrosine kinase receptor for neu-differentiation factor, is expressed in normal colonic epithelium and is up-regulated in adenomatous polyps of the colon [43]. The down regulation of cyclin D1 and c-neu mRNA abundance by SPI and WPH may at least partly explain their anti-tumorigenic properties. Similarly, Krüppel-like transcription factors have been linked to cell growth and tumorigenesis. BTEB2 (also known as Krüppel-like factor 5, KLF5, or intestinal KLF) was reported to enhance intestinal epithelia cell colony formation, cyclin D1 transcription, and cell proliferation [44]. Consistent with our results for cyclin D1, colonic BTEB2 mRNA expression was down-regulated by SPI and WPH. Aquaporin 3, a water channel highly expressed in colonic epithelium, was down-regulated by SPI and WPH. Aquaporins are thought to be induced early in colon cancer and to facilitate oncogenesis [45], therefore, dietary repression of such genes may additionally contribute to anti-tumorigenesis. The results for I-FABP and MUC3 indicated 3–4 fold decreases in transcript abundance in proximal colons of rats on SPI or WPH diets. These particular results are not easily reconciled with decreased tumorigenesis in SPI and WPH groups, since both genes are highly expressed in the normal differentiated colonic epithelium and are likely to be under-expressed in adenomas and adenocarcinomas [46]. Perhaps, these represent diet-modulated genes that are not direct participants in anti-tumorigenesis.

Gastrointestinal hormones regulate a myriad of intestinal functions including motility, absorption, digestion, cell proliferation and death, and immune response [47]. The microarray and real-time RT-PCR assays identified inductive effects of SPI and WPH on somatostatin mRNA and protein abundance. These results implicate this gene product in autocrine and paracrine mechanisms underlying colon cancer protection by SPI and WPH since somatostatin is a well-known anti-proliferative agent for colon tumor cells [48,49]. This hormone is also a negative regulator of angiogenesis [50]; this is predicted to counter tumorigenesis. It is possible that the small decrements in rat growth rates observed with lifetime SPI or WPH diets [8,9] are a consequence of this increased circulating somatostatin level. We also found decreased abundance of mRNAs encoding peptide YY (PYY) and glucagon in colons of WPH-fed rats. PYY gene expression in human colon tumors is much reduced relative to the adjacent normal tissue [51]; however, chemically-induced colon tumors in rats generally exhibit higher overall expression of PYY due to increased prevalence of PYY-positive cells, compared to normal mucosa [52,53]. PYY stimulates proliferation of intestinal epithelium [54]; therefore, an inhibition of PYY expression by dietary WPH may contribute to colon cancer-protective actions. A similar scenario might apply to colon glucagon gene expression, as this growth stimulatory peptide for colon cancer cells [55] was inhibited by WPH at the level of colon mRNA abundance.

Our data highlighted other aspects of diet and colon gene expression that warrant further study. For example, the B7 antigen (also known as CD52) mRNA was strongly down-regulated by SPI or WPH. The corresponding protein is normally expressed at high levels on cell membranes of T and B lymphocytes and monocytes; infusion with anti-CD52 antibody leads to systemic depletion of T cells [56]. The lower abundance of this transcript in non-tumor colon tissue of rats on SPI or WPH diets may reflect fewer numbers of immune cells in this tissue, as compared to CAS-fed animals. One possible interpretation of this data is that the 'normal' tissue of the CAS group has manifested an immune response, perhaps in response to increased tumorigenicity relative to SPI or WPH groups. Such an interpretation raises the prospect of a functional immuno-editing mechanism [57] occurring in this model of colon cancer and an indirect effect of diet on lymphocyte populations (via presence of tumors or tumor precursors) in the colon. An alternative mechanism is that dietary protein can directly affect the populations of lymphocytes resident in the colon, which in turn, may affect tumorigenesis. A related observation was the enhanced abundance of CD3 gamma chain transcripts in colons of SPI and WPH animals. The protein encoded by this transcript helps mediate T cell antigen receptor engagement and signaling [58]; its decreased abundance in colonic T cells of CAS-fed animals may indicate a specific immune defect [59] occurring in the CAS-fed animals after exposure to carcinogen and thereby contributing to enhanced tumorigenesis in this group.

Several microarray studies of human paired normal colon vs. colon tumors have been published [60-64]. Comparison of the present results for normal colon tissue of AOM-treated rats on different diets to the published studies for human CRC identified only a small number of common differentially expressed genes and/or gene families in common (data not shown). This small number is probably due to the fact that our study did not examine colon tumors; rather we focused on 'normal' colon tissue. In this regard, it will be interesting to examine the expression profiles of colons from animals not treated with AOM so as to more specifically correlate transcripts with diet and cancer phenotype. This study has illuminated a number of genes and gene families that may act as dietary protein-dependent modulators of oncogenesis in the rat colon. Additional studies that specifically address the functional involvement of these genes in cancer-prevention via dietary means are required to confirm the postulated roles.

## Conclusions

We have identified genes in rat colon that are differentially expressed, as a consequence of altered dietary protein, during AOM-induced oncogenesis. These are candidates for genes that sub-serve the anti-cancer effects of dietary SPI and WPH in this tissue.

## Methods

### Rats, diets and carcinogen treatment

The animals whose colons were used in the present study have been previously described [8,9]. Time-mated [gestation day (GD) 4] Sprague-Dawley rats were purchased from Harlan Industries (Indianapolis, IN), housed individually and allowed *ad libitum *access to water and pelleted food. Rats were randomly assigned to one of three semi-purified isocaloric diets made according to the AIN-93G diet formula [65] and which differed only by protein source: a) CAS (New Zealand Milk Products, Santa Rosa, CA), b) WPH (New Zealand Milk Products, Santa Rosa, CA) or c) SPI (Dupont Protein Technologies, St. Louis, MO). Offspring were weaned to the same diet as their mothers and were fed the same diets throughout the study. At 90 days of age, male offspring received s.c. injections of 15 mg/kg AOM (Ash Stevens, Detroit, MI) in saline once a week for 2 weeks. Forty weeks later, rats were euthanized, and the colon (cecum to anus) was divided into two equal segments (proximal and distal), opened longitudinally, and examined for tumors. We found that both WPH and SPI significantly decreased the colon tumor incidence [data published in [8,9]]. A representative non-tumor segment of each proximal colon (PC) was frozen in liquid nitrogen and stored at -80°C for later use. Animal care and handling were in accordance with the Institutional Animal Care & Use Committee guidelines of the University of Arkansas for Medical Sciences.

### RNA processing

Total RNA was isolated from rat proximal colons (n = 7 for each of CAS, SPI and WPH diets) using TRIzol reagent (Invitrogen, Carlsbad, CA), and further purified with the RNeasy Mini Kit (QIAGEN, Valencia, CA). To remove contaminating DNA, on-column DNA digestion with RNase-Free DNase (QIAGEN) was performed. Integrity of isolated RNAs was confirmed using the RNA 6000 Nano LabChip kit with the Agilent 2100 Bioanalyzer System (Agilent Biotechnologies, Palo Alto, CA). To reduce errors due to biological variability, RNA samples were pooled as proposed by Bakay et al [66]. Pooled RNA (equal amounts of RNA from each of 7 animals; 8 ug total) was used for cDNA synthesis using a T7-(deoxythymidine)_24 _primer and Superscript II (Life Technologies, Inc., Gaithersburg, MD). The resulting cDNA was used with the ENZO BioArray High Yield RNA Transcript labeling kit (ENZO, Farmingdale, NY) to synthesize biotin-labeled cRNA. The cRNA was purified on RNeasy spin columns (QIAGEN) and subjected to chemical fragmentation (size range of 35 to 200 bp). Three replicate cRNA targets were made in parallel starting from each RNA pool.

### Microarray procedures

Ten ug of cRNA was hybridized for 16 hours to an Affymetrix (Santa Clara, CA) rat U34A GeneChip (3 chips used per diet group), followed by incubations with streptavidin-conjugated phycoerythrin, and then with polyclonal anti-streptavidin antibody coupled to phycoerythrin. Following washing, GeneChips were scanned using an Agilent GeneArray laser scanner. Images were analyzed using Affymetrix MAS 5.0 software. Bacterial sequence-derived probes on the arrays served as external controls for hybridization, whereas the housekeeping genes β-actin and GAPDH served as endogenous controls and for monitoring the quality of the RNA target. To compare array data between GeneChips, we scaled the average of the fluorescent intensities of all probes on each array to a constant target intensity of 500.

### Bioinformatics

To validate the microarray procedure for our samples, unsupervised nearest-neighbor hierarchical clustering (Spotfire, Somerville, MA) was performed on gene expression data. The inter-chip variability test also was performed as specified in the Affymetrix data analysis manual [28]. To identify colon genes differentially expressed with SPI or WPH (control: CAS diet), multiple criteria were applied; final results are reported only for transcripts that passed all three analytical steps described below. Firstly, the t-test feature of DMT (Affymetrix) was used to identify genes whose expression was regulated (induced/repressed with P < 0.05) by SPI or WPH, and signal fold changes (FC) for these genes were calculated. Secondly, microarray data were analyzed using 'Significance of Analysis of Microarrays' (SAM, Stanford) to identify significant changes in gene expression among diet groups [67], using a false discovery rate (FDR) cutoff of 0.5%. Lastly, a pair-wise comparison survival (3 × 3) method was used to identify differentially expressed transcripts [68]. In brief, the three replicate expression profiles obtained for SPI colons were iteratively compared with the three CAS profiles (latter as baseline) in MAS 5.0 (Affymetrix), generating nine comparisons in total. Transcripts with a log ratio greater than or equal to 1 (≥2 fold change), which increased (I) in nine of nine comparisons, and which were expressed above background (i.e., called as Present) in all three SPI GeneChips, were considered to be up-regulated by SPI. Transcripts with a log ratio less than or equal to -1, were decreased (D) in nine of nine comparisons, and expressed above background (Present) in all three CAS chips were considered to be down-regulated by SPI. WPH-regulated genes were similarly identified. Genes that were independently identified by all three approaches comprised the final reported lists of differentially expressed genes (Tables 2, 3, 4, 5, 6).

### Validation of gene expression by quantitative real-time RT-PCR

One μg of total RNA from each of the 21 individual proximal colons (which comprised the original pools for the microarray experiment) was reverse-transcribed using random hexamers and MultiScribe Reverse Transcriptase in a two-step RT-PCR reaction (Applied Biosystems, Foster City, CA). Primers (Table 7) were designed using 'Primer Express' (Applied Biosystems) and were selected to yield a single amplicon; this was verified by dissociation curves and/or analysis in agarose gels. SYBR Green real-time PCR was performed with an ABI Prism 7000 Sequence Detector. Thermal cycling conditions included pre-incubation at 50°C for 2 min, 95°C for 10 min followed by 40 PCR cycles at 95°C for 15 sec and 60°C for 1 min. The relative transcript levels for each gene were calculated using the relative standard curve method (User Bulletin #2, Applied Biosystems) and normalized to the house-keeping gene β-actin. Data are reported as mean ± SEM of n = 7 animals per dietary group. Significant differences between diet groups were determined by one-way ANOVA (*P *< 0.05).

**Table 7:** Primer sequences for real-time RT-PCR

Gene	Forward primer	Reverse primer	Accession no.
Beta-actin	5'-GACGGTCAGGTCATCACTATCG-3'	5'-ACGGATGTCAACGTCACACTTC-3'	NM_031144
I-FABP	5'-AGGAAGCTTGGAGCTCATGACA-3'	5'-TCCTTCCTGTGTGATCGTCAGTT-3'	K01180
Neu Oncogene	5'-GTGGTCGTTGGAATCCTAATCAA-3'	5'-CCTTCCTTAGCTCCGTCTCTTTTA-3'	X03362
PYY	5'-AGGAGCTGAGCCGCTACTATGC-3'	5'-TTCTCGCTGTCGTCTGTGAAGA-3'	M17523
Glucagon	5'-TGGTGAAAGGCCGAGGAAG-3'	5'-TGGTGGCAAGGTTATCGAGAA-3'	K02813
Somatostatin	5'-GGAAACAGGAACTGGCCAAGT-3'	5'-TGCAGCTCCAGCCTCATCTC-3'	K02248
PAP III	5'-AAGAGGCCATCAGGACACCTT-3'	5'-CACTCCCATCCACCTCTGTTG-3'	L20869
CYP4F1	5'-CCAAGTGGAAACGGTTGATTTC-3'	5'-TCCTGGCAGTTGCTGTCAAAG-3'	M94548
GST	5'-ACTTCCCCAATCTGCCCTACTTA-3'	5'-CGAATCCGCTCCTCCTCTGT-3'	X04229
Cyclin D1	5'-TCAAGTGTGACCCGGACTGC-3'	5'-ACTTCCCCTTCCTCCTCGGT-3'	D14014
Beta defensin-1	5'-TCTTGGACGCAGAACAGATCAATA-3'	5'-TCCTGCAACAGTTGGGCTATC-3'	AF093536
H+, K+-ATPase	5'-ATTCCGCATCCCTAGACAACG-3'	5'-TCTTACTAAAGCTGGCCATGATGTT-3'	M90398
Prostaglandin D synthetase	5'-CAAGCTGGTTCCGGGAGAAG-3'	5'-TTGGTCTCACACTGGTTTTTCCTTA-3'	J04488
RBP	5'-TCGTTTCTCTGGGCTCTGGTAT- 3'	5'-TTCCCAGTTGCTCAGAAGACG-3'	M10934
Muc3	5'-AAGGTGTGAGGAAGTGATGGAGA-3'	5'-GCAGAGACCGTCGGCTTTATC-3'	U76551
BTEB1	5'-ACACTGGTCACCATCGCCAA-3'	5'-GGACTCGACCCAGATTCGGT-3'	NM_057211
BTEB2	5'-CTACTTTCCCCCATCACCACC-3'	5'-GAATCGCCAGTTTCGAAGCA-3'	AB096709

### Serum Sst

Rat serum Sst content (15 animals from each diet) was determined using the somatostatin-28 EIA kit purchased from Phoenix Pharmaceuticals Corporation (Belmont, California).

## Authors' contributions

RX performed the microarray and real-time PCR experiments, conducted the data analysis, and participated in drafting the manuscript. TMB designed and oversaw the animal component of the study. FAS designed the analytical and overall approaches to the study, supervised the project, and drafted the manuscript. All authors read and approved the final manuscript.
